# Hereditary angiooedema and psychological stress: an exploratory study

**DOI:** 10.1186/2045-7022-5-S1-O6

**Published:** 2015-03-11

**Authors:** Anna Galante, Maria Francesca Freda, Maria Bova, Raffaella De Falco, Raffaele De Luca Picione, Gianni Marone, Angelica Petraroli, Livia Savarese, Gerarda Siani, Paolo Valerio, Massimo Triggiani

**Affiliations:** 1 University of Naples Federico II, Department of Neuroscience, Naples; Italy; 2 University of Naples Federico II, Department of Humanities, Naples; Italy; 3 University of Naples Federico II, Division of Allergy and Clinical Immunology, Naples; Italy; 4 University of Salerno, Division of Allergy and Clinical Immunology, Salerno, Italy

## 

Hereditary Angiooedema (HAE) is characterized by a deficit or by a malfunctioning of C1- 1nh; its symptoms vary greatly from one individual to another. Some studies suggest that some of the attacks of HAE may be triggered or affected by stress and emotional states (Zotter et al., 2014), however this link has never been examined in depth. Moreover, recent research has highlighted the influence of the neurological correlatives of stress in the activation of the compliment cascade, a system already compromised in those suffering from HAE [1][2].

## Aims

With this pilot study we intend to explore the connection between stress, emotional states and the variability of the attacks, in a sample of 11 individuals aged between 4 and 17 with HAE, together with their parents. It is a preliminary study, which aims to explore whether emotions have a direct or indirect influence on the development of HAE.

## Methods

We adopt a multi method approach, using widely -used, internationally recognized tests, listed below:

- Semi-structured interviews with parents to explore their interpretation on the variability of the attacks;

- CBCL to exclude cases of psychopathology;

- CLES to evaluate the perceived level of stress;

- AQC on alexithymia;

- LEAS-C on emotional awareness;

- TEMAS on the functions of personality;

- A diary of symptoms, noting the frequency, intensity and the location of the attacks.

## Results

Although 92% of parents believed that emotional states played an important role in the triggering of attacks, the young patients were unable or had difficulty articulating their emotional states, more in general with their processes of *emotion regulation* (De Steno et al., 2013). This may well be connected to the high levels of stress experienced by nearly all the patients seen in the CLES test. These high levels of stress, in 9 out of 11 cases, correlated with the frequency of the attacks. Finally, from the CBCL results no personality disorder emerged connected to HAE.

## Conclusions

We believe that by encouraging skills aimed at expressing emotions caused by stress, prove useful in the treatment and management of individuals suffering from HAE. The results of this pilot study highlighted the need for further research on the role of psychological factors connected to HAE.

## References

[B1] Burns V “Complement cascade Activation after an acute psychological stress task” Psychosomatic Medicine 2008 70 387 396 10.1097/PSY.0b013e31816ded22 18480187

[B2] De Steno D Gross JJ Kubzansky L "Affective Science and Health:The Importance of Emotion and Emotion Regulation” Health Psychology 2013 32 5 474 486 23646831 10.1037/a0030259

